# Sulfur Metabolizing Microbes Dominate Microbial Communities in Andesite-Hosted Shallow-Sea Hydrothermal Systems

**DOI:** 10.1371/journal.pone.0044593

**Published:** 2012-09-07

**Authors:** Yao Zhang, Zihao Zhao, Chen-Tung Arthur Chen, Kai Tang, Jianqiang Su, Nianzhi Jiao

**Affiliations:** 1 State Key Laboratory of Marine Environmental Sciences, Xiamen University, Xiamen, China; 2 Institute of Marine Geology and Chemistry, National Sun Yat-Sen University, Taiwan, ROC; 3 Key Laboratory of Urban Environment and Health, Institute of Urban Environment, Chinese Academy of Sciences, Xiamen, China; Argonne National Laboratory, United States of America

## Abstract

To determine microbial community composition, community spatial structure and possible key microbial processes in the shallow-sea hydrothermal vent systems off NE Taiwan’s coast, we examined the bacterial and archaeal communities of four samples collected from the water column extending over a redoxocline gradient of a yellow and four from a white hydrothermal vent. Ribosomal tag pyrosequencing based on DNA and RNA showed statistically significant differences between the bacterial and archaeal communities of the different hydrothermal plumes. The bacterial and archaeal communities from the white hydrothermal plume were dominated by sulfur-reducing *Nautilia* and *Thermococcus*, whereas the yellow hydrothermal plume and the surface water were dominated by sulfide-oxidizing *Thiomicrospira* and *Euryarchaeota* Marine Group II, respectively. Canonical correspondence analyses indicate that methane (CH_4_) concentration was the only statistically significant variable that explains all community cluster patterns. However, the results of pyrosequencing showed an essential absence of methanogens and methanotrophs at the two vent fields, suggesting that CH_4_ was less tied to microbial processes in this shallow-sea hydrothermal system. We speculated that mixing between hydrothermal fluids and the sea or meteoric water leads to distinctly different CH_4_ concentrations and redox niches between the yellow and white vents, consequently influencing the distribution patterns of the free-living *Bacteria* and *Archaea*. We concluded that sulfur-reducing and sulfide-oxidizing chemolithoautotrophs accounted for most of the primary biomass synthesis and that microbial sulfur metabolism fueled microbial energy flow and element cycling in the shallow hydrothermal systems off the coast of NE Taiwan.

## Introduction

Hydrothermal vents occur over a wide depth range, from the intertidal to the abyssal [1]. Deep-sea hydrothermal vents have received a great deal of attention since their discovery, with a focus on origin of life studies. However, shallow-sea hydrothermal vents, at depths of 200 meters or less, have been studied less. In hydrothermal fluids, reduced compounds, such as hydrogen sulfide (H_2_S), methane (CH_4_) and hydrogen (H_2_), are the most important for biological processes and, thus, active sulfur and CH_4_ cycling are usually considered to occur in either deep-sea or shallow-sea hydrothermal systems. Besides chemotrophy based on sulfur and/or CH_4_, phototrophy may be another important process in the shallow-sea vent fields since sunlight and hydrothermal energy co-support these systems [1], [2]. Furthermore, unlike the deep-sea hydrothermal vent where the effect of venting is restricted to a very narrow zone near the discharge, the volcanic fluids affect not only the near-bottom water layer, but also the surface in the shallow-sea vent fields, thus affecting whole ecosystems [1].

Such an area of shallow marine hydrothermal venting was observed approximately one kilometer east of the Kueishantao Islet, near the southern end of the Okinawa Trough (Fig. S1). A cluster of shallow (<30 m in depth) yellow and white hydrothermal vents emit a mass of elemental sulfur which is precipitated on the seafloor forming high chimneys. Gas discharges from these vents are dominated by carbon dioxide (CO_2_) (>92%) with small amounts of H_2_S [3]. Elemental analysis and isotopic characteristics reveal that the origin of the hydrothermal fluid is complex. The fluid is composed mainly of three parts: the deep magmatic matter, the sea water, and meteoric water from the Kueishantao Islet [4]. As the deep magmatic matter rises, the volatiles separate from the magma. They continue to rise and meet and mix with the descending sea water and meteoric water to form the end member of the hydrothermal fluid. A large amount of H_2_S and sulfur dioxide from magma degassing are emitted into the fluid, and the interactions of the fluid with the subsurface igneous rocks (andesite) of the Kueishantao Islet leach out low concentrations of trace metals and lanthanides from these rocks [5]. When the fluid meets seawater, elemental sulfur and other sulfur compounds were formed by oxidation. Thus, elemental sulfur and sulfur compounds are the predominant products from the hydrothermal fluid. In addition, other reductive gases from the magma, such as CH_4_ and H_2_, are included in the hot fluid [6], [7].

Another pronounced feature of this shallow-sea hydrothermal field is the presence of a greater enrichment in oxygen and biogenic elements, e.g. nitrogen, phosphorus and silicon, brought by the meteoric water [1]. Varying ratios of magmatic, sea and meteoric water create a great variety of chemical conditions in the shallow-water hydrothermal systems. Microorganisms that thrive in this habitat should possess nutritional requirements and overall metabolic pathways that are ideally suited to the shallow-sea hydrothermal ecosystem and they mediate much of the transfer of elements and energy that occurs along the redox gradient from vents to surface water. However, so far, no data related to the microbial community composition, spatial structure and interaction between hydrothermal fluid geochemistry and microbial community are reported for such a shallow marine hydrothermal venting field off NE Taiwan’s coast.

In the present study, by pyrosequencing the V1–3 hypervariable region of the bacterial 16S rRNA gene and the V6 region of the archaeal 16S rRNA gene from both DNA and RNA (i.e., complementary DNA [cDNA]) pools, we constructed and analyzed a total of 32 bacterial and archaeal DNA- and RNA-based tag libraries from eight samples from the two shallow-sea hydrothermal vents: four from a yellow and four from a white hydrothermal vent. Our sampling bracketed the whole water column depth ranging over both a redoxocline and gradients in major geochemical parameters. An attempt was made to link these geochemical parameters to a characterization of the microbial community structure and to address the biogeochemical processes that are mediated by microorganisms supported the energy flow and element cycling in such shallow-sea hydrothermal systems.

## Results

### Physio-chemical Parameters

**Figure 1:**
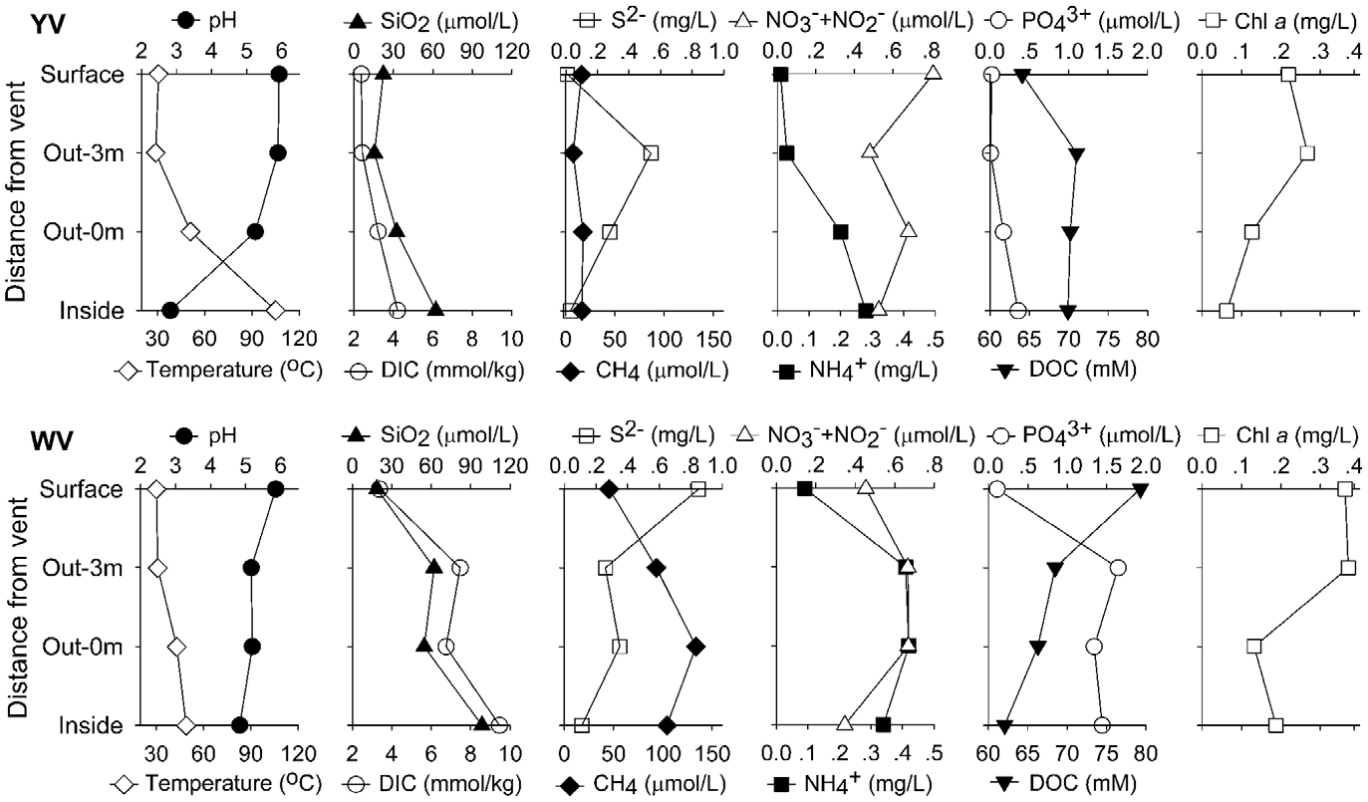
Chemical parameters along the vertical gradients of the yellow (YV) and white (WV) hydrothermal vent plumes.

We collected water samples inside a yellow (YV_Inside) and white (WV_Inside) hydrothermal vent (in the seafloor ∼1 m deep inside the vent), at 0 m (YV_Out-0 m; WV_Out-0 m) and ∼3 m (YV_Out-3 m; WV_Out-3 m) above the vents, and from the surface water immediately above the vents (YV_Surface; WV_Surface). Vertical profiles of the chemical parameters for the two vents are given in Fig. 1. The yellow hydrothermal vent had a temperature of 105°C and a pH value as low as 2.82, whereas the white vent had a lower temperature (49°C) and a higher pH value (4.83). The salinity was nearly oceanic (data not shown). The silicon dioxide (SiO_2_) and dissolved inorganic carbon (DIC) concentrations increased along the gradient from the surface to the vents. CH_4_, ammonium (NH_4_^+^) and phosphate (PO_4_^3−^) concentrations were also, overall, higher at the vents than in the surface water. Compared to deep-sea vents, sulfide (S^2−^) concentration was relatively lower in this shallow vent system, whereas relatively higher concentrations of chlorophyll *a* and dissolved organic carbon (DOC) [12] were found here. Compared to the yellow vent field, CH_4_, DIC, SiO_2_, PO_4_^3−^ and NH_4_^+^ concentrations were higher at the white vent. CH_4_ concentrations were even one order of magnitude higher and had a stronger increasing trend from the surface to the vent, thus suggesting a higher redox potential for the white hydrothermal plume.

### Library Statistics

PCR reactions were successful with all bacterial templates, but with the archaeal primers two (a DNA template from YV_Out-0 m and a cDNA template from YV_Inside) were not successful (Table S1 and S2). A total of 101,751 bacterial and 63,164 archaeal reads passed the pipeline filters that removed reads that contained errors, or were of poor quality and chloroplast origin, or could not be confidently assigned to a correct domain. Sequences were clustered into operational taxonomic units (OTUs) with cut-off values set at 0.03, 0.05 and 0.10. Non-parametric coverage, phylotype richness estimators and diversity indices (ACE, Chao1 and Shannon) of each library are shown in Tables S1 and S2. The diversity indices showed a higher level of species richness for bacterial RNA pools than for the corresponding DNA pools, except for the sample from YV_Inside. Most of the archaeal RNA-based libraries also appeared to be more diverse than their DNA-based counterparts, but the difference between the richness indices were not significant. The same conclusion was supported by rarefaction analysis (Fig. S2 and S3).

Rarefaction curves of the number of OTUs versus sampling effort were generated using two threshold values for OUT −97% and 90%. They all showed an increasing slope, indicating that sampling did not reach saturation in our tag libraries (Fig. S2 and S3). Overall, bacterial diversity was much greater than archaeal diversity in all samples. The rank logarithmic abundance of the OTUs in each of our bacterial and archaeal libraries is plotted in Fig. S4 and S5. The archaeal DNA- and RNA-based libraries were dominated by a few abundant OTUs. The first 20 most abundant ≥97% and ≥90% OTUs accounted for about 80% and 90% of the total archaeal community, respectively. However, in the bacterial DNA- and RNA-based libraries, the first 100 most abundant ≥97% and ≥90% OTUs accounted for 30–60% and 52–92% of the total community, respectively.

### Community Comparisons

**Figure 2:**
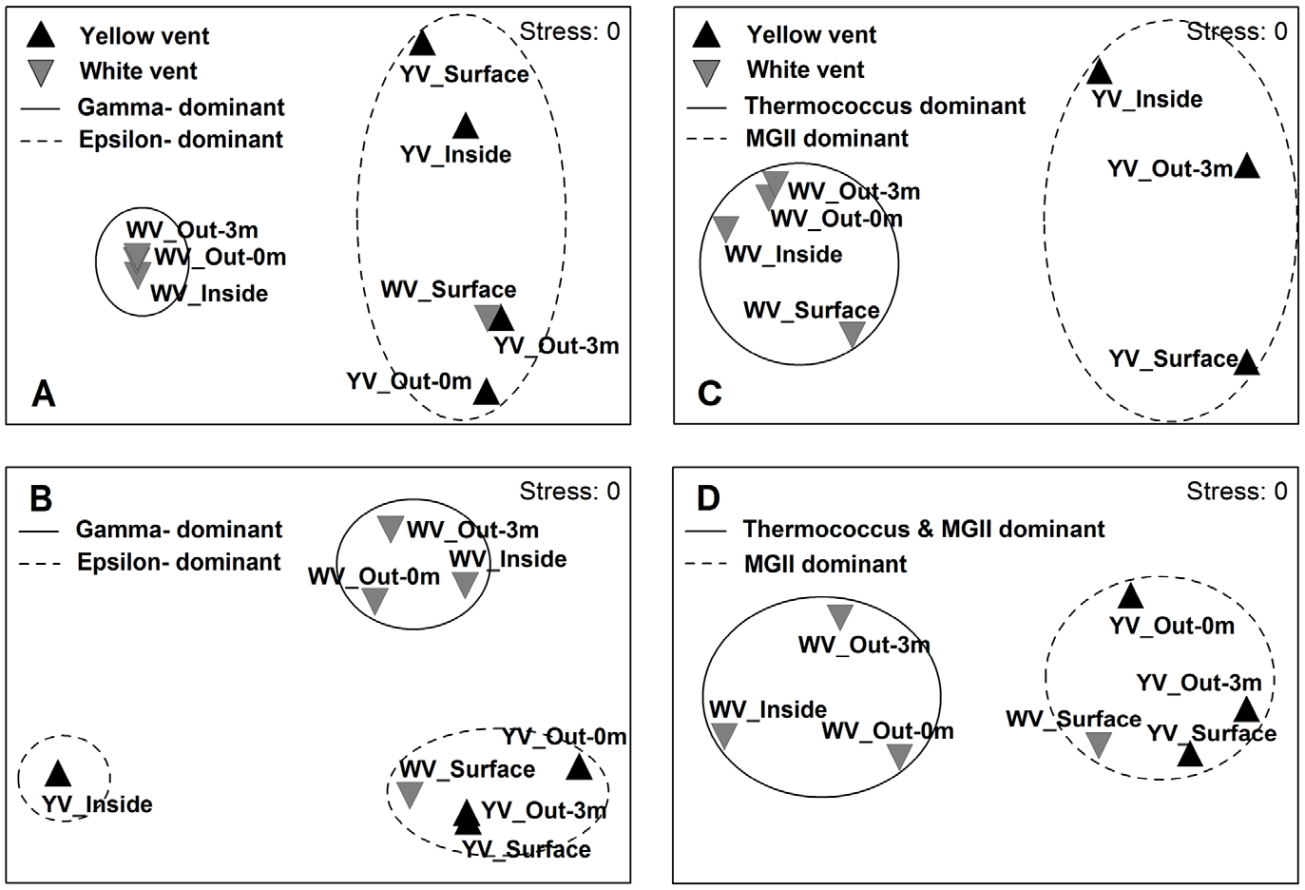
NMDS ordination of the Bray-Curtis similarities in bacterial or archaeal community composition. (A) bacterial DNA-based libraries. (B) bacterial RNA-based libraries. (C) archaeal DNA-based libraries. (D) archaeal RNA-based libraries. Each point represents an individual sample. Circles separate different clusters. ANOSIM analysis shows that the observed cluster patterns are significant.

Non-metric multidimensional scaling (NMDS) based on the relative abundance of OTUs was built from Bray-Curtis similarities to discriminate the bacterial or archaeal community composition between different samples. Cluster analysis showed that the archaeal DNA-based libraries separated into one cluster containing communities of the yellow hydrothermal vent at 56% similarity and one cluster of the white vent at 81% similarity. The bacterial DNA-based libraries had a similar cluster pattern with the exception of WV_Surface in the cluster of the yellow vent (Fig. 2A and C). Two clusters of the yellow and white vents were identified at the 55% and 83% similarity level, respectively. The NMDS of the archaeal RNA-based libraries also produced two clusters that correspond to the yellow and white vents at 73% and 77% similarity, respectively, but WV_Surface was included in the cluster of the yellow vent. The NMDS of the bacterial RNA-based libraries produced three clusters, one cluster including communities of the yellow vent and WV_Surface at 62% similarity, one cluster of the white vent at 59% similarity and one cluster containing the sole community from YV_Inside (Fig. 2B and D). Statistical significances determined by the analysis of similarity (ANOSIM) test were observed in all cluster analyses (global *R* = 1, *P*<0.05). Similarity percentage (SIMPER) analysis revealed that the differences in bacterial or archaeal community composition between clusters were explained by the dominant groups.

**Figure 3:**
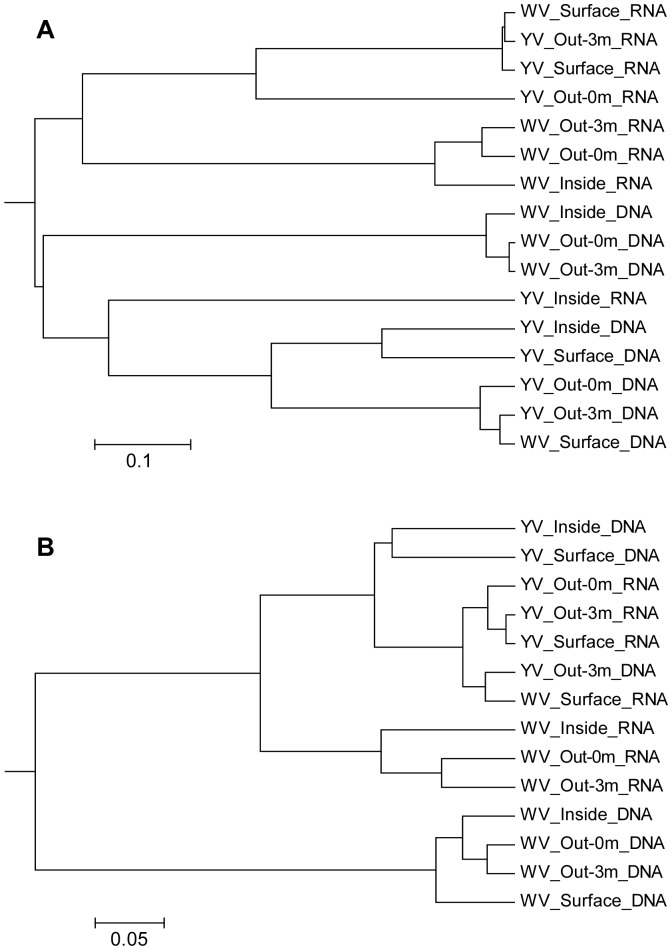
UPGMA trees constructed from Thetayc distances between bacterial libraries (A) and Bray-Curtis distances between archaeal libraries (B).

The unweighted pair group method with arithmetic mean (UPGMA) trees constructed from Thetayc distances (MOTHUR) for bacterial libraries and Bray-Curtis distances (VAMPS) for archaeal libraries showed the same results as the NMDS analysis. Furthermore, the bacterial DNA- and RNA-based libraries from all samples were partitioned in separated clusters, except for the RNA-based library from YV_Inside, which was included in the DNA cluster (Fig. 3A). For archaeal communities, the DNA- and RNA-based libraries from the yellow vent were joined together into one cluster. However, the archaeal DNA-based libraries from the white hydrothermal vent were most alike, and the RNA-based libraries were also more alike, with the exception of the RNA-based library from WV_Surface which was in the cluster of the yellow vent (Fig. 3B).

### Relationship between Community Structure and Environmental Factors

**Figure 4:**
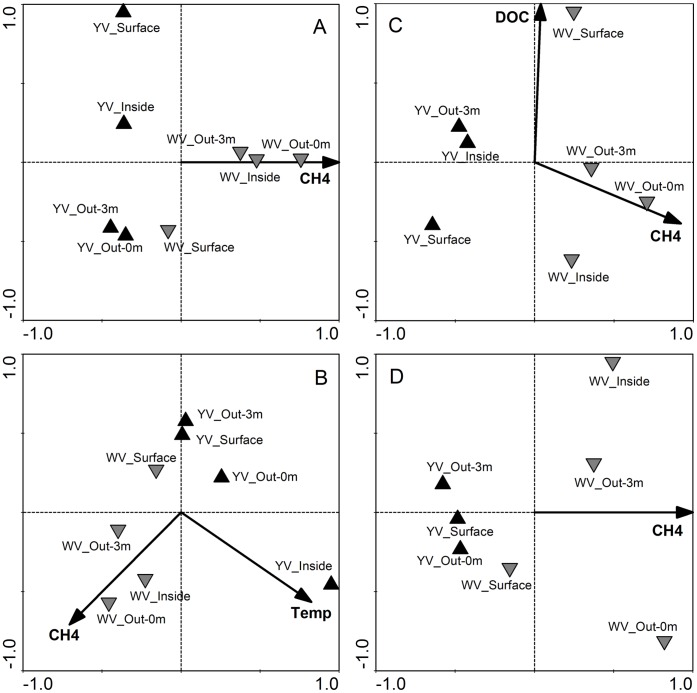
CCA (A, C and D) or RDA (B) analyses of bacterial or archaeal communities. (A) bacterial DNA-based libraries. (B) bacterial RNA-based libraries. (C) archaeal DNA-based libraries. (D) archaeal RNA-based libraries. Each point represents an individual sample. Arrowheads represent statistically significant environmental variables explaining the observed patterns (*P* = 0.002). CH4: methane. Temp: temperature. DOC: dissolved organic carbon.

The relationship between bacterial or archaeal assemblage structure and environmental properties was assessed by correlating the two distance matrices with a Mantel test. The similarity matrix of environmental factors included all available variables. The bacterial assemblage structure was significantly correlated with the environmental factors (DNA-based libraries: *r* = 0.674, *P* = 0.007; RNA-based libraries: *r* = 0.474, *P* = 0.003). However, for the archaeal assemblage, only the composition of the RNA-based libraries significantly correlated with the environmental variables (*r* = 0.674, *P* = 0.007). Among all the environmental variables, the combination of variables “best explaining” the community pattern was obtained using biota-environment stepwise analysis (BVSTEP) in PRIMER. Both CH_4_ and NH_4_ emerged as highly significant explanatory variables for the bacterial DNA-based libraries, while CH_4_, NH_4_, temperature and pH were responsible for the RNA-based libraries. For archaeal communities, CH_4_, NH_4_, DOC and DIC concentrations appeared to be the key factors in determining the composition of the DNA-based libraries, and CH_4_, DIC and SiO_2_ concentrations were the best combination of explanatory variables for the RNA-based libraries. CH_4_ appeared to be the most important factor. The canonical correspondence analysis (CCA) (Fig. 4A, C and D) or redundancy analysis (RDA) (Fig. 4B) yielded high similarity patterns with the NMDS analysis, and revealed that CH_4_ concentration was the only statistically significant variable that explains the all community cluster patterns (*P* = 0.002) (Fig. 4). Furthermore, temperature (*P* = 0.002) together with CH_4_ separated the three clusters in the RDA analysis of the bacterial RNA-based libraries (Fig. 4B). DOC concentration (*P* = 0.002) was another significant factor for the archaeal DNA-based pattern (Fig. 4C). In the four CCA/RDA models of the bacterial DNA- and RNA-based libraries and the archaeal DNA- and RNA-based libraries, the environmental variables explained 45%, 77%, 57% and 43% of the total variance in the community composition, respectively.

### Phylogenetic Identification

**Figure 5:**
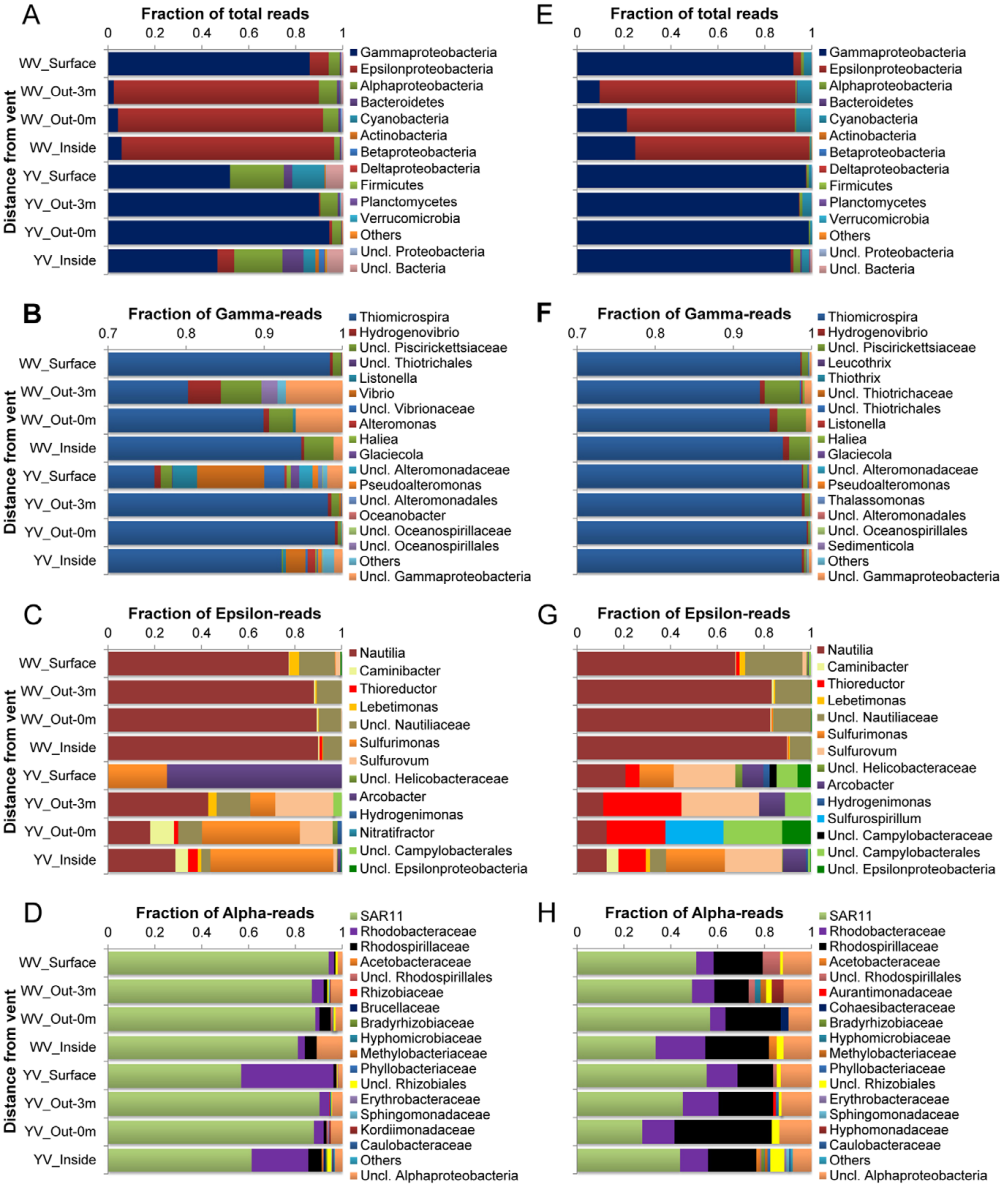
Distribution of tags by phylogenetic taxa among the bacterial DNA- (A-D) and RNA-based libraries (E-H). (A) and (E) Relative abundances of bacterial phyla or classes in total tags of each library. (B) and (F) Relative abundance of bacterial genera in total gammaproteobacterial tags. (C) and (G) Relative abundance of bacterial genera in total epsilonproteobacterial tags. (D) and (H) Relative abundance of bacterial families in total alphaproteobacterial tags.

**Figure 6:**
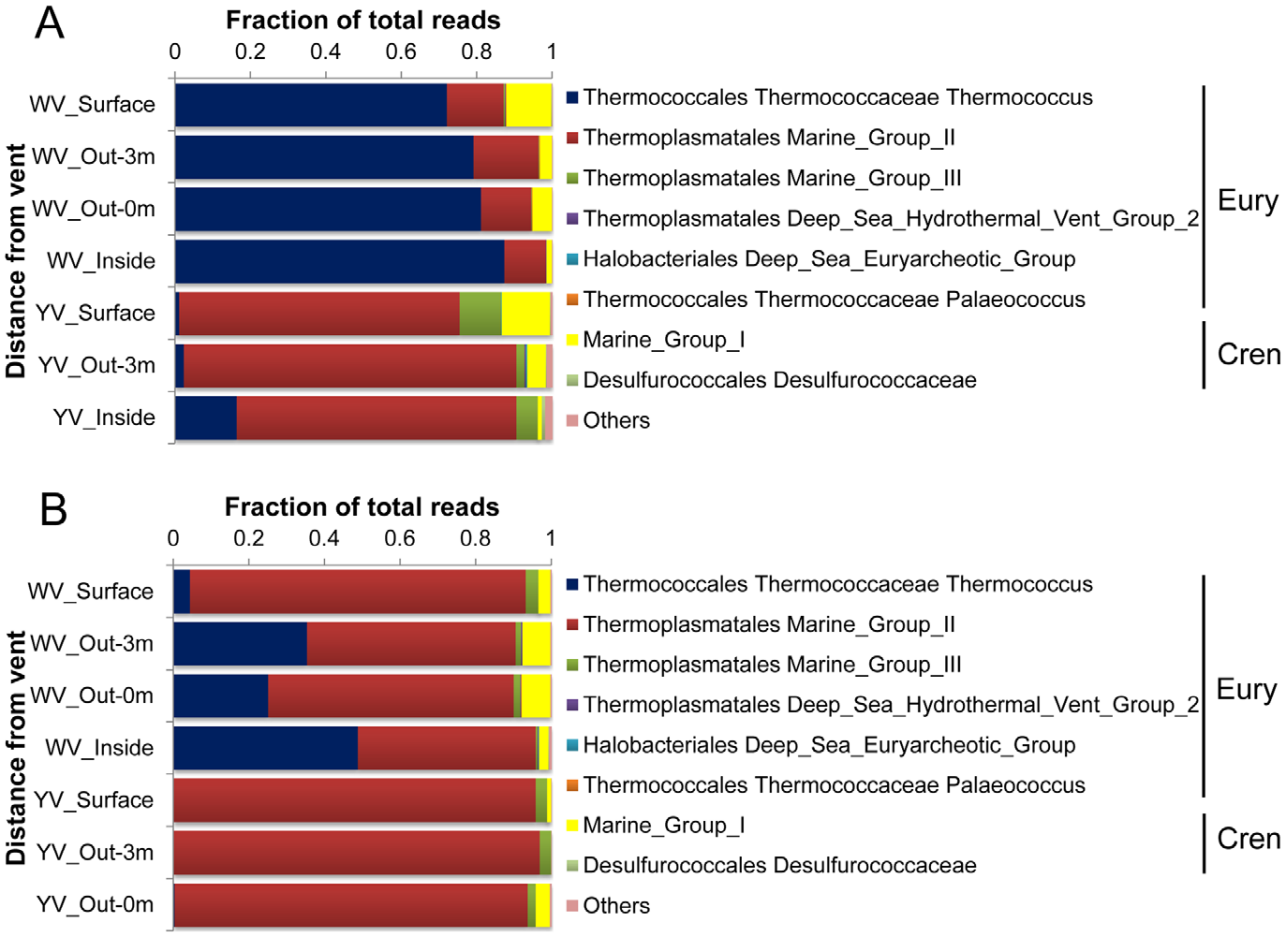
Distribution of tags by phylogenetic taxa among the archaeal DNA- (A) and RNA-based libraries (B).

Ribotypes of tags were identified phylogenetically and grouped by phylum, class, family or genus. The relative abundance of a given phylogenetic group in all tags or class level is given in Fig. 5 and 6. *Proteobacteria* dominated every bacterial library (mostly >90%), and the *Gammaproteobacteria* or *Epsilonproteobacteria* were the most abundant groups. The *Gammaproteobacteria* dominated the communities from the yellow hydrothermal vent and the surface of the white vent (DNA-based libraries: 46–94%; RNA-based libraries: 91–98%), while the *Epsilonproteobacteria* dominated the other three communities from the white vent (DNA-based libraries: 87–91%; RNA-based libraries: 72–84%) (Fig. 5A and E). Within the *Gammaproteobacteria*, the mesophilic sulfide-oxidizing *Thiomicrospira* comprised the major fraction amounting to 76–99% of the total gammaproteobacterial tags in the DNA-based libraries and to 93–99% in the RNA-based libraries (Fig. 5B and F). Within the *Epsilonproteobacteria*, the moderately thermophilic *Nautilia* accounted for 77–90% and 67–90% in the DNA- and RNA-based libraries from the white hydrothermal vent, respectively, but only accounted for 0–43% and 11–21% in the DNA- and RNA-based libraries from the yellow hydrothermal vent (Fig. 5C and G). Other significant members within the *Epsilonproteobacteria* included the *Caminibacter*, *Thioreductor*, *Lebetimonas*, *Sulfurimonas*, *Sulfurovum*, *Arcobacter*, *Hydrogenimonas*, *Nitratifractor* and *Sulfurospirillum*. The *Alphaproteobacteria* were, overall, dominated by the SAR11 clade in the DNA- (57–94%) and RNA-based libraries (28–57%) (Fig. 5D and H). In addition, the *Rhodobacteraceae* and *Rhodospirillaceae* were also relatively abundant within the *Alphaproteobacteria*. Another significant group was the *Cyanobacteria*. It accounted for 0.1–14% and 0.5–6% of total tags in the DNA- and RNA-based libraries, respectively (Fig. 5A and E). In comparison, the *Betaproteobacteria*, *Deltaproteobacteria*, *Actinobacteria*, *Firmicutes*, *Planctomycetes* and *Verrucomicrobia* tags were more likely to appear as ‘rare’ taxa. Only a few sequences (<1% of total tags in each library with the exception of <7% for the two DNA-based libraries) could not be identified to at least the phylum level.

The *Euryarchaeota* dominated every archaeal library (>86%). *Thermococcus* (72–87% of total tags) and Marine Group II (74–88%) were most abundant in the DNA-based libraries from the white and yellow hydrothermal vents, respectively (Fig. 6A). In the RNA-based libraries, Marine Group II dominated the communities from the yellow vent and the surface of the white vent (89–97% of total tags), while the other three communities from the white vent were co-dominated by Marine Group II (47–65%) and *Thermococcus* (25–49%) (Fig. 6B). Marine Group III also accounted for a significant proportion of the DNA-based libraries from the yellow vent (2–11% of total tags) and the RNA-based libraries from the two vents (0.5–4%). Marine Group I comprised the major fraction of the *Crenarchaeota*, accounting for 1–13% and 0.06–8% of total tags in all DNA- and RNA-based libraries, respectively. Extremely rare sequences affiliated with the Deep-sea Hydrothermal Vent Euryarcheotic Group 2, the Deep-sea Euryarcheotic Group, *Palaeococcus*, *Desulfurococcaceae* and *Methanothermococcus* were recovered sporadically at the two vent fields.

## Discussion

The richness estimates from pyrosequencing showed much richer DNA and RNA pools than did the analyses of clone libraries from these samples (data not shown), revealing many rare taxa. The rarefaction curves (Fig. S2 and S3) indicated that, even with OTUs constructed by clustering sequences within a 10% distance, none of our libraries sampled a pool to saturation. This meant that many taxa were still missed, especially in the case of the bacterial libraries. However, the rarefaction curves seemed to be inconsistent with the distribution of tags among phylogenetic taxa (Fig. 5 and 6), which indicated high dominance of abundant taxa and low richness of communities. SIMPER analysis revealed surprisingly high diversity within these dominant genera with several OTUs, thus suggesting that many species or ecotypes were responsible for the high diversity of the bacterial and archaeal libraries. It is possible that these species or ecotypes filled different ecological niches in such a hydrothermal habitat that has a great variety of chemical conditions [8].

Cluster analyses based on Bray-Curtis similarity revealed a distinct community spatial structure. Overall, the bacterial or archaeal libraries separated into two clusters that correspond to the two vents. The most striking difference between the two clusters was the dominance of *Epsilonproteobacteria* and *Thermococcus* in the white hydrothermal system (except for the surface water) versus *Gammaproteobacteria* and *Euryarchaeota* Marine Group II in the yellow hydrothermal system for the bacterial and archaeal communities, respectively.

*Epsilonproteobacteria* are known to be key players in sulfidic habitats, catalyzing elemental sulfur reduction and oxidation reactions [13]. So it is not surprising that *Epsilonproteobacteria* are consistently shown to be the most numerically abundant bacteria in hydrothermal vent environments [9], [13]–[16]. The most abundant tag within the *Epsilonproteobacteria* in our libraries was related to the moderately thermophilic sulfur-reducing *Nautilia*, which is capable of chemolithoautotrophic growth on molecular H_2_ or formate as the electron donor and elemental sulfur as the electron acceptor, producing H_2_S [17]. A model species, *N. profundicola*, contains all the genes necessary for life under extreme conditions such as anaerobic, sulfur, H_2_- and CO_2_-rich environments with fluctuating redox potentials and temperatures [18].

*Gammaproteobacteria* were the most abundant sequences observed in the yellow vent and the surface samples. The most abundant tag within *Gammaproteobacteria* in our libraries was related to the sulfide-oxidizing *Thiomicrospira*, which is one of the most abundant culturable sulfur oxidizers and is normally dominant at shallow vents [1]. Members of the genus *Thiomicrospira* are chemolithoautotrophic bacteria that use reduced sulfur compounds (thiosulfate, tetrathionate, sulfur and sulfide) as electron donors and obtain carbon from CO_2_. *Thiomicrospira* is also a dominant community member of hydrothermal vent sites in the Mid-Atlantic Ridge [19].

The *Alphaproteobacteria* accounted for a few of the communities in our libraries. Overall, the most abundant tag was related to the SAR11 clade. Genomic and metagenomic data suggested that members of the ubiquitous and abundant SAR11 clade are deficient in assimilatory sulfate reduction genes, which are normally necessary for all aerobic marine bacteria to supply sulfur for biosynthesis [20]. So SAR11 depends on exogenous sources of reduced sulfur for growth. This could explain the presence of the SAR11 clade as a significant group in the shallow-sea hydrothermal system. Another explanation is that SAR11 have been entrained in the vent fluids due to mixing with seawater where it is typically very numerous. The co-presence of photosynthetic and chemolithotrophic microorganisms is a unique feature of shallow-sea hydrothermal vents [1], [2] and, as we predicted, *Cyanobacteria* were recovered from our libraries, and were a significant component in the RNA pools.

*Thermococcus* are thermophilic, anaerobic, organotrophic, sulfur-reducing archaeon. In their growth reactions, they require elemental sulfur as electron acceptors, producing H_2_S and CO_2_. Thus, they probably play an important role in organic matter breakdown, with very high rates of sulfur reduction [1]. This could explain why DOC concentration separated the two clusters in the CCA analysis of the archaeal DNA-based libraries, together with CH_4_, as a significant factor. *Thermococcus*, as a thermophile, is usually found to thrive at temperatures between 60 and 105°C and in a pH range of 5 to 9. In our present study they were abundantly present in the white hydrothermal vent where the pH was relatively more favorable but the temperature lower than in the yellow vent (Fig. 1). We speculated that *Thermococcus* might have thrived deep inside the white vent where optimal growth temperature occurred, or that some unknown species and ecotypes could have grown at temperatures below 60°C. The Marine Group II *Euryarchaea* were another main archaeal group in the shallow-sea hydrothermal system. Their dominance suggested that efficient mixing between hydrothermal fluids and seawater had occurred, especially for the yellow vent field, since they are widely distributed and quite abundant at times, especially near the ocean’s surface [21].

The distribution of bacterial tags among phyla, classes, families or genera based on the DNA pool was broadly consistent with the sequence data from their RNA-based counterparts, especially for the abundant taxa. Sequence libraries constructed from cDNA should interrogate the active moiety of a microbial community [22], [23], since RNA pools are governed by the rapid rate of intracellular RNA turnover and the short lifetime of extracellular RNA compared to DNA [24]. Thus, a comparison of our bacterial DNA- and RNA-based sequence data suggested that more active OTUs were also more abundant. Moreover, the overwhelming dominance of *Nautilia* (60–70%) and *Thiomicrospira* (90–98%) in the RNA pools of the white (excluding the surface zone) and yellow hydrothermal systems implied that sulfur reduction that was mediated mostly by *Nautilia* and sulfide oxidization that was performed primarily by *Thiomicrospira* might have occurred respectively in the two vent fields (Fig. 5). However for archaeal communities, the main difference between the DNA and RNA pools was the dominance of *Thermococcus* versus the co-dominance of *Thermococcus* and the Marine Group II in the white hydrothermal system (Fig. 6). This suggested that *Thermococcus* was less active, although it was abundant in the communities, compared to Marine Group II. *Thermococcus*, as mentioned above, was quite limited to extreme environmental conditions of temperature, pH and redox potential. Thus, mixing between hydrothermal fluids and seawater could have disturbed their niches, consequently resulting in their cells becoming less active.

At the hydrothermal vents, reducing fluids are injected into oxic waters, generating hydrothermal plumes with sharp chemical gradients, where microorganisms mediate much of the transfer of elements and energy [18]. Although methanogens and methanotrophs are common inhabitants of most vent fields [25], the results of pyrosequencing showed their almost complete absence at the two vent fields we investigated. Only extremely rare sequences affiliating with methanogenic *Archaea* (18 sequences in total) and methanotrophic *Bacteria* (20 in total) were recovered from our libraries, even when elevated CH_4_ concentrations were generated in the white hydrothermal system, suggesting that the microbial communities might have been less tied to the CH_4_ cycle in the shallow-sea hydrothermal system. However, our CCA/RDA analyses indicated that CH_4_ concentration was the only statistically significant variable that explains the all community cluster patterns (*P* = 0.002) (Fig. 4). We speculated that the mixing between hydrothermal fluids and sea or meteoric water led to distinctly different CH_4_ concentrations and ecological niches between the yellow and white vents, consequently influencing the distribution patterns of the free-living *Bacteria* and *Archaea*. Less mixing between the hydrothermal fluids and oxygenated seawater, which was implied by high CH_4_ concentrations, allowed the fluid to remain in a reducing sate and allowed the sulfide reduction reaction to occur. On the contrary, because of the higher emitting speed (data not shown), more efficient mixing of seawater might have occurred within the yellow vent field, resulting in relatively lower CH_4_ concentrations and an oxygenated environment, so that sulfide oxidation was an energetically favorable reaction in the enhanced mixing zones. So, along the vertical gradient from the vent to the surface water, we also saw the community succession from the dominance of sulfur-reducing *Nautilia* to sulfide-oxidizing *Thiomicrospira* in the white hydrothermal system. We concluded that sulfide-oxidizing and sulfur-reducing chemolithoautotrophs accounted for most of the primary biomass synthesis, and that microbial sulfur metabolism appeared to be a central driving force for the microbial energy flow and element cycling of the shallow hydrothermal ecosystem off the NE coast of Taiwan. Also, it is possible that the microbes attached to the minerals may tell a different story and they deserve to be examined in the future.

## Materials and Methods

### Study Sites and Sampling

Two hydrothermal vents, one yellow and one white, were observed at depths of about 8.5 and 16.2 m, respectively. The vents were identified by scuba divers and their positions were located using a Global Positioning System (GPS). Sealed, custom made, polyvinylchloride tubes were used by divers to collect the sample waters inside the vents (in the seafloor ∼1 m deep inside the vent), at 0 m and ∼3 m above the vents, and from the surface immediately above the vents. In total, eight samples were collected from the two vents. All necessary permits were obtained for the described field studies.

Two liter samples for DNA or RNA analysis were filtered through 3 µm and 0.2 µm pore-size polycarbonate filters (Millipore) at a pressure <0.03 MPa on board. Samples for RNA extraction were finished within 30 min and stored in 2 mL RNase-free tubes with RNA*later* RNA stabilization solution (Ambion). All filters were immediately frozen and stored in liquid nitrogen until DNA or RNA extraction.

### Biogeochemical Analysis

In-situ temperatures were measured by scuba divers with a thermocouple. A Guildline salinometer (Autosal 8400B) was used to measure conductivity, which was then converted to salinity, and pH values were determined using a Radiometer PHM-85 pH meter at 25°C. Values for nitrate as well as nitrite were obtained using the pink azo dye method [26], [27] with a flow injection analyzer; PO_4_^3−^ was determined using the molybdenum blue method; and SiO_2_ was measured employing the silicomolybdenum blue method [26], also with a flow injection analyzer. The NH_4_^+^ and S^2−^ concentrations were measured using the indophenol method [28] and the methylene blue method, respectively, with an HACH DR/890 colorimeter. Dissolved CH_4_ was measured by gas chromatography using the gas-stripping method [29]. DIC was measured using a Dissolved Inorganic Analyzer (Model AS-C3) with a precision of 0.1%, and CO_2_ certified reference materials were used to assess the accuracy of sample measurements. DOC concentration was measured using the method of high temperature catalytic oxidation following the removal of inorganic carbon by acidification and oxygen purging, using a high TOCII analyzer (Elementar, Germany). The accuracy of the measurements was verified with Low Carbon Water and Deep Sea Water (from Dr. D. A. Hansell, University of Miami). Chlorophyll *a* concentration was determined using the acetone extraction method and a laboratory fluorometer (Varian Eclipse).

### DNA and RNA Extraction

Genomic DNA extractions were performed with an MO-BIO UltraClean kit following the manufacturer’s protocols. The quality and quantity of DNA were checked with a NanoDrop device (ND-2000, Thermo Fisher) and kept at −80°C until use. RNA was extracted using an RNeasy Mini kit (Qiagen) according to the protocol of the manufacturer. DNA digestion was performed with an RNase-Free DNase Set (Qiagen) during RNA purification. Reverse transcription reactions were performed using the SuperScript RT-PCR system with random hexamers (Invitrogen) to synthesize first-strand cDNA.

### PCR Amplification of the V1–3 Region of Bacterial and the V6 Region of Archaeal 16S rRNA Genes

Universal primers 27F and 534R were used for PCR amplification of the V1–3 hypervariable regions of bacterial 16S rRNA genes [30]. The forward primer 27F (shown in bold letters in the following) contained the 454 Life Sciences primer A sequence (shown in underlined letters in the following) at the 5′ end: 5′-CGTATCGCCTCCCTCGCGCCATCAGA**GAGTTTGATCCTGGCTCAG**-3′. The reverse primer 517R contained the 454 Life Sciences primer B sequence at the 5′ end and a unique 10 bp error-correcting Golay barcode used to tag each PCR product (designated by NNNNNNNNNN): 5′-CTATGCGCCTTGCCAGCCCGCTCAGNNNNNNNNNN**ATTACCGCGGCTGCTGG**-3′. PCR reactions were carried out in triplicate 25-µL reactions with 0.4 µM forward and reverse primers, 20–50 ng of template DNA or cDNA, and 2× Premix Taq (TaKaRa). Negative controls without a template were included for each barcoded primer pair to test for reagent contamination. For PCR with the cDNA template, RNA samples without the RT step were also used as controls to test for residual DNA in the RNA preparations. Thermal cycling consisted of initial denaturation at 94°C for 3 min followed by 30 cycles of denaturation at 94°C for 30 seconds, annealing at 55°C for 45 seconds, and extension at 72°C for 45 seconds, with a final extension of 7 min at 72°C. Replicate amplicons were pooled and run in a 1% agarose gel. Bands of the expected size were excised and purified with an agarose gel DNA purification kit (TaKaRa). The quality and quantity of the products were checked with a NanoDrop device (ND-2000, Thermo Fisher).

The archaeal hypervariable V6 region of the 16S rRNA gene was amplified using primers containing a unique 8 bp error-correcting barcode (designated by NNNNNNNNNN) with “CA” (shown in italics) inserted as a linker between the barcode and rRNA primer: 958archF 5′-NNNNNNNN*CA*AATTGGANTCAACGCCGG-3′, 1048archR-major 5′-NNNNNNNN*CA*CGRCGGCCATGCACCWC-3′ and 1048archR-minor 5′-NNNNNNNN*CA*CGRCRGCCATGYACCWC-3′ under the conditions previously described [10]. PCR reactions were carried out in triplicate 50-µL reactions, and the amplification protocol was the same as for bacteria.

### Amplicon Quantitation, Pooling, and Pyrosequencing

PCR products were quantified using a Quant-iT PicoGreen dsDNA kit (Invitrogen) according to the manufacturer’s instructions. Assays were carried out using 2 µL of cleaned PCR product in a total reaction volume of 200 µL in black 96-well microplates. Fluorescence was measured on a FlexStation 3 Microplate Reader (Molecular Devices) using 480/520-nm excitation/emission. Equimolar amounts of the PCR amplicons were mixed in a single tube. The ethanol precipitation process was carried out for the final pool of DNA in order to remove amplification primers and reaction buffer [31]. The final concentration of the purified amplicon mixture was determined using a NanoDrop spectrophotometer (ND-2000, Thermo Fisher). Pyrosequencing was carried out using a 454 Genome Sequencer GS-FLX Titanium instrument (Roche-454 Life Sciences) at the Chinese National Human Genome Center (Shanghai, China).

### Quality Control of Sequencing and Sequence Analysis

The criteria previously described [11] were used to assess the quality of sequence reads. We eliminated sequences that contained more than one ambiguous nucleotide (N), that did not have a complete barcode and primer at one end, or were shorter than 150 bp for bacteria or 50 bp for archaea after removal of the barcode and primer sequences. The remaining sequences were assigned to samples by examining the barcode. For bacterial V1–3 region sequences, each trimmed sequence without the barcode and primer sequences was classified using the Ribosomal Database Project (RDP) Naïve Bayesian Classifier with a minimum support threshold of 50% and RDP taxonomic nomenclature.

The archaeal V6 region sequences were processed using the visualization and analysis of microbial population structure (VAMPS) pipeline with improved filtering and clustering as previously described [11], [32]–[34]. Taxonomical identification was assigned to the tags using the rRNA indexing algorithm Global Assignment of Sequence Taxonomy (GAST) [11] based on the SILVA database [35]. The GAST methodology is freely available through the VAMPS website (http://vamps.mbl.edu). All sequences obtained for this study have been deposited in the NCBI Sequence Read Archive (SRP011316).

### Library Analysis and Community Comparisons

Libraries of sequences and OTUs were further analyzed in MOTHUR [36]. Sequences were clustered into OTUs with a cut-off value set at 0.03, 0.05 and 0.10. Based on OTU assignment, library richness and diversity estimates (ACE, Chao1, Shannon) were calculated using MOTHUR’s *summary.single* routine. Rarefaction curves were calculated for the eight samples at a 0.03 and 0.10 distance cutoff using *rarefaction.single*.

NMDS was used to determine the similarity between samples with PRIMER [37]. Bray-Curtis similarities were calculated on the OTU relative abundance matrices. The similarities are presented in a multidimensional space by plotting more similar samples closer together [38], [39]. The ANOSIM [40] function in the PRIMER program was used to test for the significance of the differences in community composition among various NMDS clusters. One-way ANOSIM analysis with 999 permutations was performed based on the Bray-Curtis similarities matrices. Dendrograms relating the similarity in community membership and structure were also generated using MOTHUR with the *tree.shared* command. The same results were obtained as those with the NMDS analysis. SIMPER analysis [41] in PRIMER was used to identify which organisms were responsible for the differences observed in community composition after taxonomy was assigned to each OTU.

### Statistical Analysis of the Correlations between Community Structure and Environmental Patterns

The Mantel test was used to determine the relationships between community structure (based on the relative abundance of all OTUs) and environmental factors. BVSTEP in PRIMER was performed to find the combination of environmental variables that best explain the community pattern by maximizing a rank correlation between the community (Bray-Curtis) and environmental (Euclidean distance) similarity matrices [37], [41]. All the available environmental variables (normalized using z-score transformation) were imported into PRIMER for BVSTEP analysis and the Mantel test.

CCA or RDA was used to further analyze the variations in the bacterial or archaeal assemblages under the constraint of environmental factors with Canoco software [42]. The RDA was chosen when the maximum gradient length of detrended correspondence analysis was shorter than 3.0, otherwise CCA was chosen [43]. The null hypothesis that the bacterial or archaeal assemblage was independent of environmental parameters was tested using constrained ordination with a Monte Carlo permutation test (499 permutations). The significant explanatory parameters (*P*<0.05) without multicollinearity (variance inflation factor <20) [44] were obtained for the community structure and plotted in Fig. 4.

## Supporting Information

Figure S1 **Map of study area and location of the shallow marine hydrothermal vents.** YV, yellow hydrothermal vent; WV, white hydrothermal vent.
(TIF)

Figure S2 **Bacterial rarefaction analysis for each sample.** The curves were generated at 97% and 90% DNA or cDNA sequence identity.
(TIF)

Figure S3 **Archaeal rarefaction analysis for each sample.** The curves were generated at 97% and 90% DNA or cDNA sequence identity.
(TIF)

Figure S4 **Bacterial rank logarithmic abundance of OTUs at two sequence similarity levels (97% and 90%) in each of DNA- and RNA-based libraries.**
(TIF)

Figure S5 **Archaeal rank logarithmic abundance of OTUs at two sequence similarity levels (97% and 90%) in each of DNA- and RNA-based libraries.**
(TIF)

Table S1 **Similarity-based OTUs, species richness and diversity estimates of bacterial communities.**
(DOC)

Table S2 **Similarity-based OTUs, species richness and diversity estimates of archaeal communities.**
(DOC)

## References

[1] Tarasov VG, Gebruk AV, Mironov AN, Moskalev LI (2005) Deep-sea and shallow-water hydrothermal vent communities: two different phenomena? Chem Geol 224: 5–39.

[2] Maugeri TL, Bianconi G, Canganella F, Danovaro R, Gugliandolo C, et al (2010) Shallow hydrothermal vents in the southern Tyrrhenian Sea. Chem Ecol 26: 285–298.

[3] Chen C-TA, Zeng Z, Kuo F-W, Yang TF, Wang B-J, et al (2005) Tide-influenced acidic hydrothermal system offshore NE Taiwan. Chem Geol 224: 69–81.

[4] Liu C-H, Wang X-M, Zeng Z-G, Yin X-B, Chen C-TA, et al (2010) Origin of the hydrothermal fluid of the shallow sea near Kueishantao Island. Marine Sciences (Chinese) 34: 61–68.

[5] Zeng Z-G, Liu C-H, Chen C-tA, Yin X-b, Chen D-G, et al (2007) Cause of formation of sulfur chimneys in the hydrothermal fields near Kueishantao islet of NE Taiwan. Sci China Ser D (Chinese) 37: 1134–1140.

[6] Li Z-X (2009) Submarine volcano near Kueishantao islet. Science Development (Chinese) 437: 40–47.

[7] Liu C-H, Wang X-Y, Zhang C-H (2007) Judging chemical reactions formed native sulfur with equilibrium constant in seafloor hydrothermal activity off Kueishantao at northeast of Taiwan island. Marine Sciences (Chinese) 31: 61–64.

[8] Flores GE, Campbell JH, Kirshtein JD, Meneghin J, Podar M, et al (2011) Microbial community structure of hydrothermal deposits from geochemically different vent fields along the Mid-Atlantic Ridge. Environ Microbiol 13: 2158–2171. 21418499 10.1111/j.1462-2920.2011.02463.x

[9] Huber JA, Cantin HV, Huse SM, Welch DB, Sogin ML, et al (2010) Isolated communities of *Epsilonproteobacteria* in hydrothermal vent fluids of the Mariana Arc seamounts. FEMS Microbiol Ecol 73. 10.1111/j.1574-6941.2010.00910.x 20533947

[10] Huber JA, Welch DBM, Morrison HG, Huse SM, Neal PR, et al (2007) Microbial population structures in the deep marine biosphere. Science 318: 97. 17916733 10.1126/science.1146689

[11] Sogin ML, Morrison HG, Huber JA, Welch DM, Huse SM, et al (2006) Microbial diversity in the deep sea and the underexplored ‘rare biosphere’. Proc Natl Acad Sci USA 103: 12115–12120. 16880384 10.1073/pnas.0605127103 PMC1524930

[12] Yang L, Hong H, Guo W, Chen C-TA, Pan P-I, et al (2012) Absorption and fluorescence of dissolved organic matter in submarine hydrothermal vents off NE Taiwan. Mar Chem 128–129: 64–71.

[13] Campbell BJ, Engel AS, Porter ML, Takai K (2006) The versatile ε-proteobacteria: key players in sulphidic habitats. Nat Rev Microbiol 4: 458–468. 16652138 10.1038/nrmicro1414

[14] Longnecker K, Reysenbach AL (2001) Expansion of the geographic distribution of a novel lineage of *ε*-proteobacteria to a hydrothermal vent site on the Southern East Pacific Rise. FEMS Microbiol Ecol 35: 287–293. 11311439 10.1111/j.1574-6941.2001.tb00814.x

[15] Nakagawa S, Takai K, Inagaki F, Hirayama H, Nunoura T, et al (2005) Distribution, phylogenetic diversity and physiological characteristics of *ε*-proteobacteria in a deep-sea hydrothermal field. Environ Microbiol 7: 1619–1632. 16156735 10.1111/j.1462-2920.2005.00856.x

[16] Opatkiewicz AD, Butterfield DA, Baross JA (2009) Individual hydrothermal vents at Axial Seamount harbor distinct subseafloor microbial communities. FEMS Microbiol Ecol 70: 413–424. 19796141 10.1111/j.1574-6941.2009.00747.x

[17] Smith JL, Campbell BJ, Hanson TE, Zhang CL, Cary SC (2008) *Nautilia profundicola* sp. nov., a thermophilic, sulfur-reducing *epsilonproteobacterium* from deep-sea hydrothermal vents. Int J Syst Evol Micr 58: 1598–1602. 10.1099/ijs.0.65435-0 18599701

[18] Campbell BJ, Smith JL, Hanson TE, Klotz MG, Stein LY, et al (2009) Adaptations to submarine hydrothermal environments exemplified by the genome of *Nautilia profundicola* . PLoS Genet 5(2): e1000362. 19197347 10.1371/journal.pgen.1000362 PMC2628731

[19] Muyzer G, Teske A, Wirsen CO, Jannasch HW (1995) Phylogenetic relationships of Thiomicrospira species and their identification in deep-sea hydrothermal vent samples by denaturing gradient gel electrophoresis of 16S rDNA fragments. Arch Microbiol 164: 165–172. 7545384 10.1007/BF02529967

[20] Tripp HJ, Kitner JB, Schwalbach MS, Dacey JWH, Wilhelm LJ, et al (2008) SAR11 marine bacteria require exogenous reduced sulphur for growth. Nature 452: 741–744. 18337719 10.1038/nature06776

[21] DeLong EF (2006) Archaeal mysteries of the deep revealed. Proc Natl Acad Sci USA 103: 6417–6418. 16618931 10.1073/pnas.0602079103 PMC1458900

[22] Frias-Lopez J, Shi Y, Tyson GW, Coleman ML, Schuster SC, et al (2008) Microbial community gene expression in ocean surface waters. Proc Natl Acad Sci USA 105: 3805–3810. 18316740 10.1073/pnas.0708897105 PMC2268829

[23] Gaidos E, Rusch A, Ilardo M (2011) Ribosomal tag pyrosequencing of DNA and RNA from benthic coral reef microbiota: community spatial structure, rare members and nitrogen-cycling guilds. Environ Microbiol 13: 1138–1152. 21176054 10.1111/j.1462-2920.2010.02392.x

[24] Novitsky JA (1986) Degradation of dead microbial biomass in a marine sediment. Appl Environ Microbiol 52: 504–509. 16347148 10.1128/aem.52.3.504-509.1986 PMC203563

[25] Takai K, Nakagawa T, Reysenbach A-L, Hoek J (2006) Microbial ecology of Mid-Ocean Ridges and Back-Arc Basins. In: Christie DM, Fisher CR, Sang-Mook L, Givens S, editors. Back-Arc spreading systems: geological, biological, chemical, and physical interactions. Washington: American Geophysical Union. 185–213.

[26] Pai SC, Yang CC, Riley JP (1990) Formation kinetics of the pink azo dye in the determination of nitrite in natural waters. Analyt Chim Acta 232: 345–349.

[27] Parsons TR, Maita Y, Lalli CM (1984) A manual of chemical and biological methods for seawater analysis. Oxford: Pergamon Press. 173 p.

[28] Grasshoff K, Ehrhardt M, Kremling K (1983) Methods of seawater analysis. Weinheim: Verlag Chemie. 419 p.

[29] Swinnerton JW, Linnenbom VJ (1967) Determination of C1 to C4 hydrocarbons in seawater by gas chromatography. J Gas Chromatogr 5: 570–573.

[30] Yildirim S, Yeoman CJ, Sipos M, Torralba M, Wilson BA, et al (2010) Characterization of the fecal microbiome from non-human wild primates reveals species specific microbial communities. PLoS One 5: e13963. 21103066 10.1371/journal.pone.0013963 PMC2980488

[31] Dominguez-Bello MG, Costello EK, Contreras M, Magris M, Hidalgo G, et al (2010) Delivery mode shapes the acquisition and structure of the initial microbiota across multiple body habitats in newborns. Proc Natl Acad Sci USA 107: 11971–11975. 20566857 10.1073/pnas.1002601107 PMC2900693

[32] Huse SM, Dethlefsen L, Huber JA, Welch DM, Relman DA, et al (2008) Exploring microbial diversity and taxonomy using SSU rRNA hypervariable tag sequencing. PLoS Genet 4: e1000255. 19023400 10.1371/journal.pgen.1000255 PMC2577301

[33] Huse SM, Huber JA, Morrison HG, Sogin ML, Welch DM (2007) Accuracy and quality of massively parallel DNA pyrosequencing. Genome Biol 8: R143. 17659080 10.1186/gb-2007-8-7-r143 PMC2323236

[34] Huse SM, Welch DM, Morrison HG, Sogin ML (2010) Ironing out the wrinkles in the rare biosphere through improved OTU clustering. Environ Microbiol 12: 1889–1898. 20236171 10.1111/j.1462-2920.2010.02193.x PMC2909393

[35] Pruesse E, Quast C, Knittel K, Fuchs BM, Ludwig W, et al (2007) SILVA: a comprehensive online resource for quality checked and aligned ribosomal RNA sequence data compatible with ARB. Nucleic Acids Res 35: 7188–7196. 17947321 10.1093/nar/gkm864 PMC2175337

[36] Schloss PD, Westcott SL, Ryabin T, Hall JR, Hartmann M, et al (2009) Introducing mothur: open-source, platform-independent, community-supported software for describing and comparing microbial communities. Appl Environ Microbiol 75: 7537–7541. 19801464 10.1128/AEM.01541-09 PMC2786419

[37] Clarke KR, Gorley RN (2001) PRIMER v5. User manual/tutorial. Plymouth: PRIMER-E Ltd. 91 p.

[38] Kruskal JB (1964) Multidimensional scaling by optimizing goodness of fit to a non-metric hypothesis. Psychometrika 29: 1–27.

[39] Kruskal JB (1964) Nonmetric multidimensional scaling - anumerical method. Psychometrika 29: 115–129.

[40] Clarke KR (1993) Non-parametric multivariate analyses of changes in community structure. Aust J Ecol 18: 117–143.

[41] Clarke KR, Warwick RM (2001) Change in marine communities: an approach to statistical analysis and interpretation. Plymouth: PRIMER-E Ltd. 172 p.

[42] Ter-Braak CJF (1989) CANOCO - an extension of DECORANA to analyze species-environment relationships. Hydrobiologia 184: 169–170.

[43] Lepš J, Šmilauer P (2003) Multivariate analysis of ecological data using CANOCO. Cambridge: Cambridge University Press. 269 p.

[44] Ter-Braak CJF (1986) Canonical correspondence analysis: a new eigenvector technique for multivariate direct gradient analysis. Ecology 67: 1167–1179.

