# Supplementary figures and images for: Sulfur Metabolizing Microbes Dominate Microbial Communities in Andesite-Hosted Shallow-Sea Hydrothermal Systems
Source: PLoS One. 2012 Sep 7;7(9):e44593. doi: 10.1371/journal.pone.0044593 (PMC3436782; doi:10.1371/journal.pone.0044593)

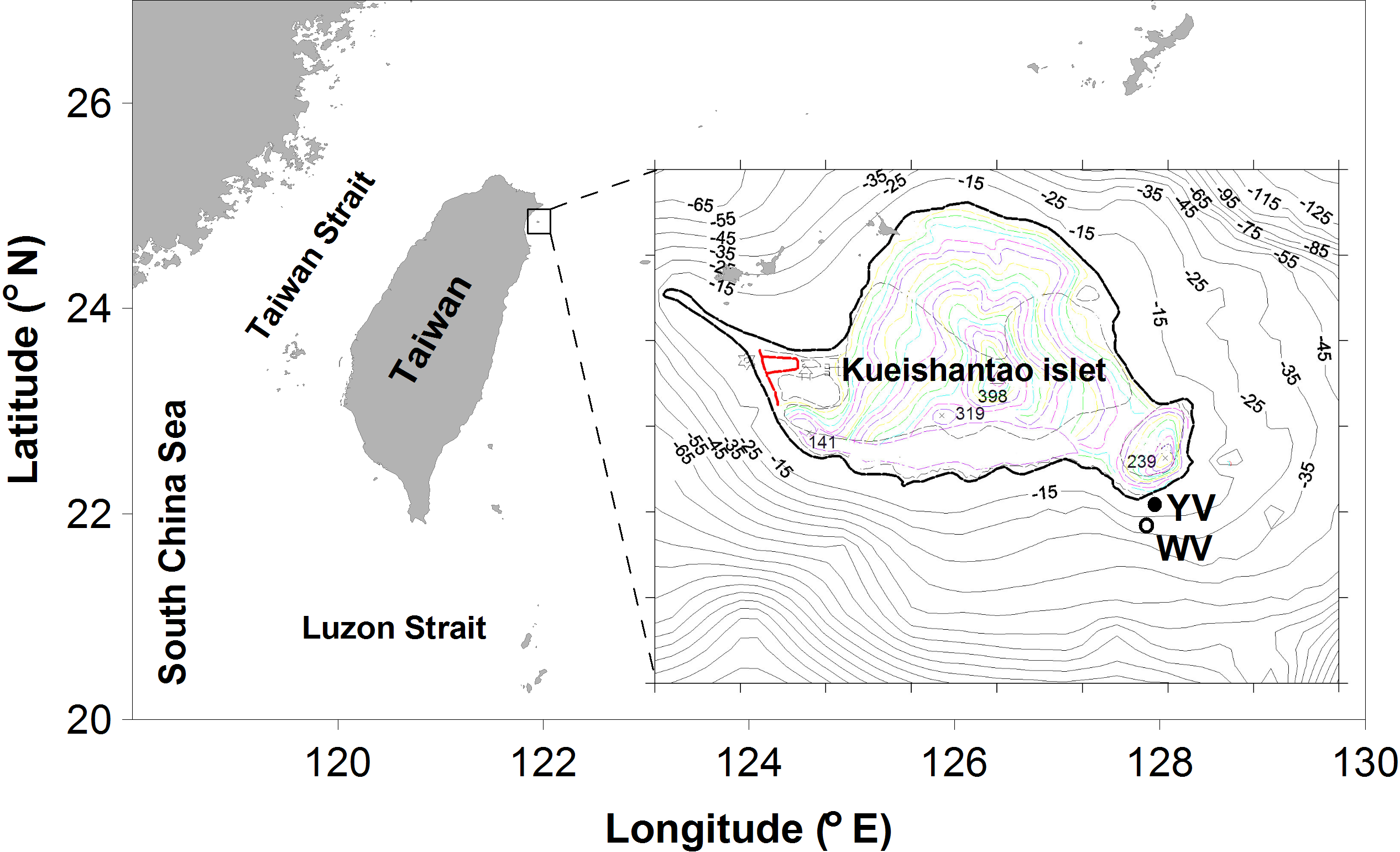

Supplement: Figure S1 — Map of study area and location of the shallow marine hydrothermal vents. YV, yellow hydrothermal vent; WV, white hydrothermal vent. (TIF) [file pone.0044593.s001.tif]

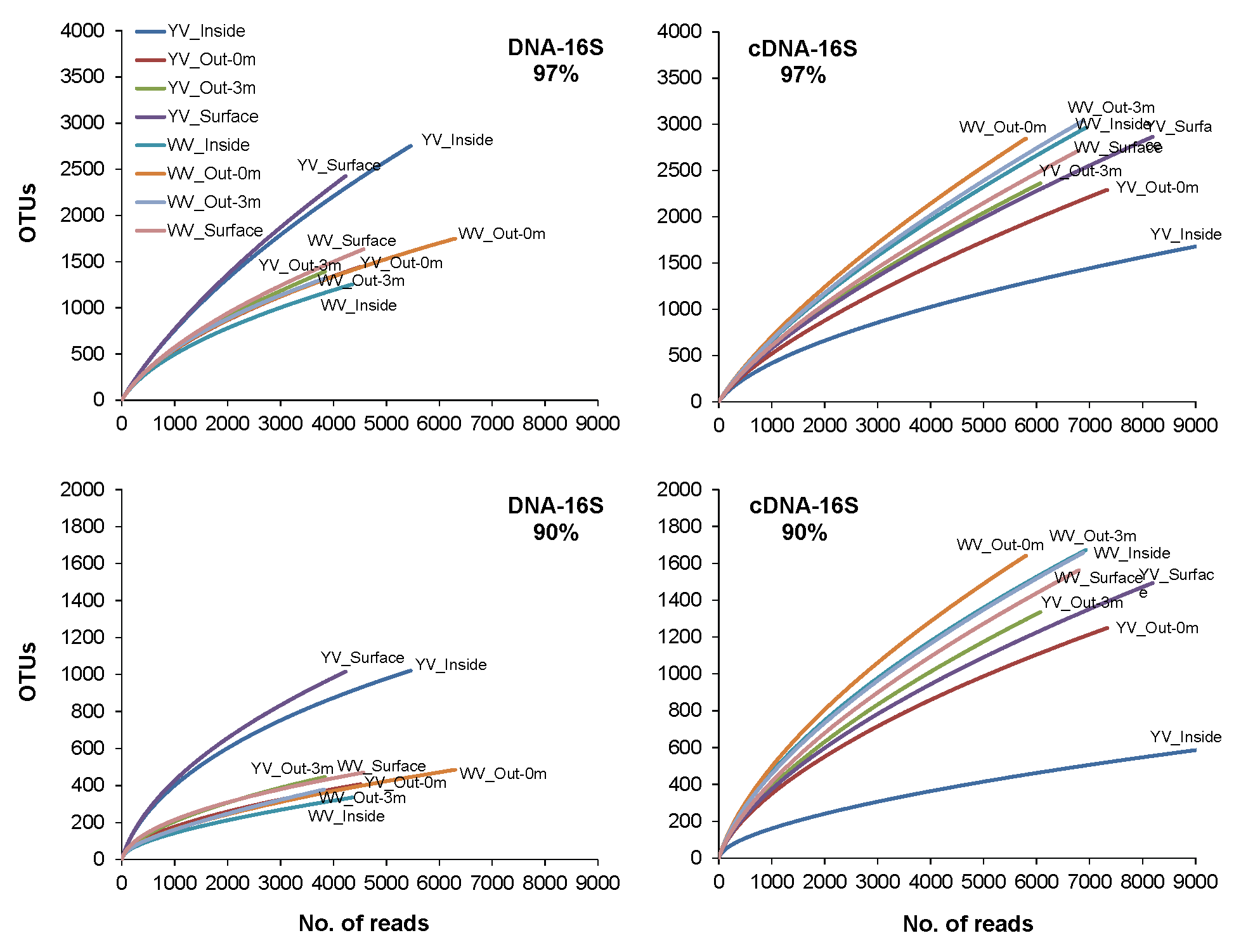

Supplement: Figure S2 — Bacterial rarefaction analysis for each sample. The curves were generated at 97% and 90% DNA or cDNA sequence identity. (TIF) [file pone.0044593.s002.tif]

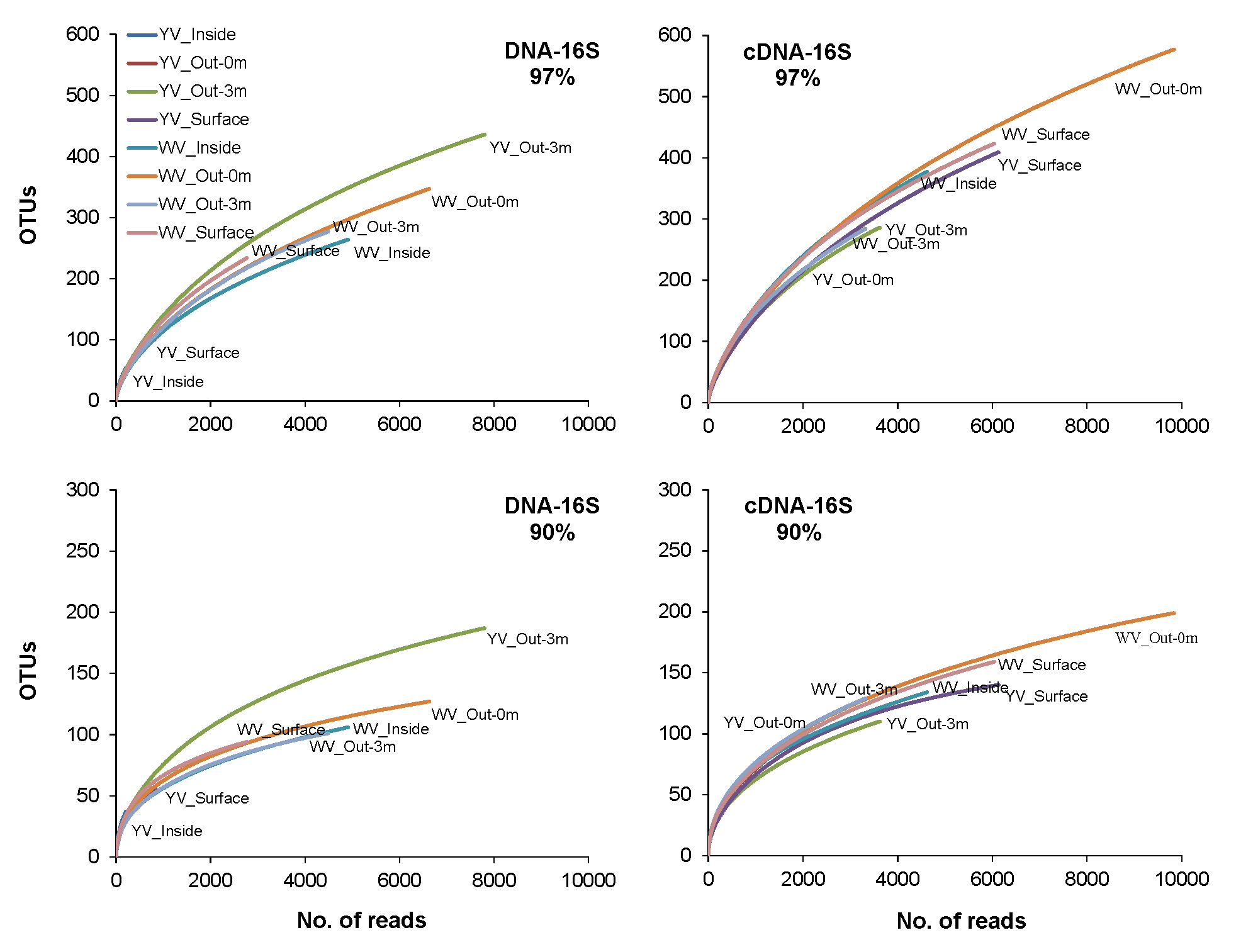

Supplement: Figure S3 — Archaeal rarefaction analysis for each sample. The curves were generated at 97% and 90% DNA or cDNA sequence identity. (TIF) [file pone.0044593.s003.tif]

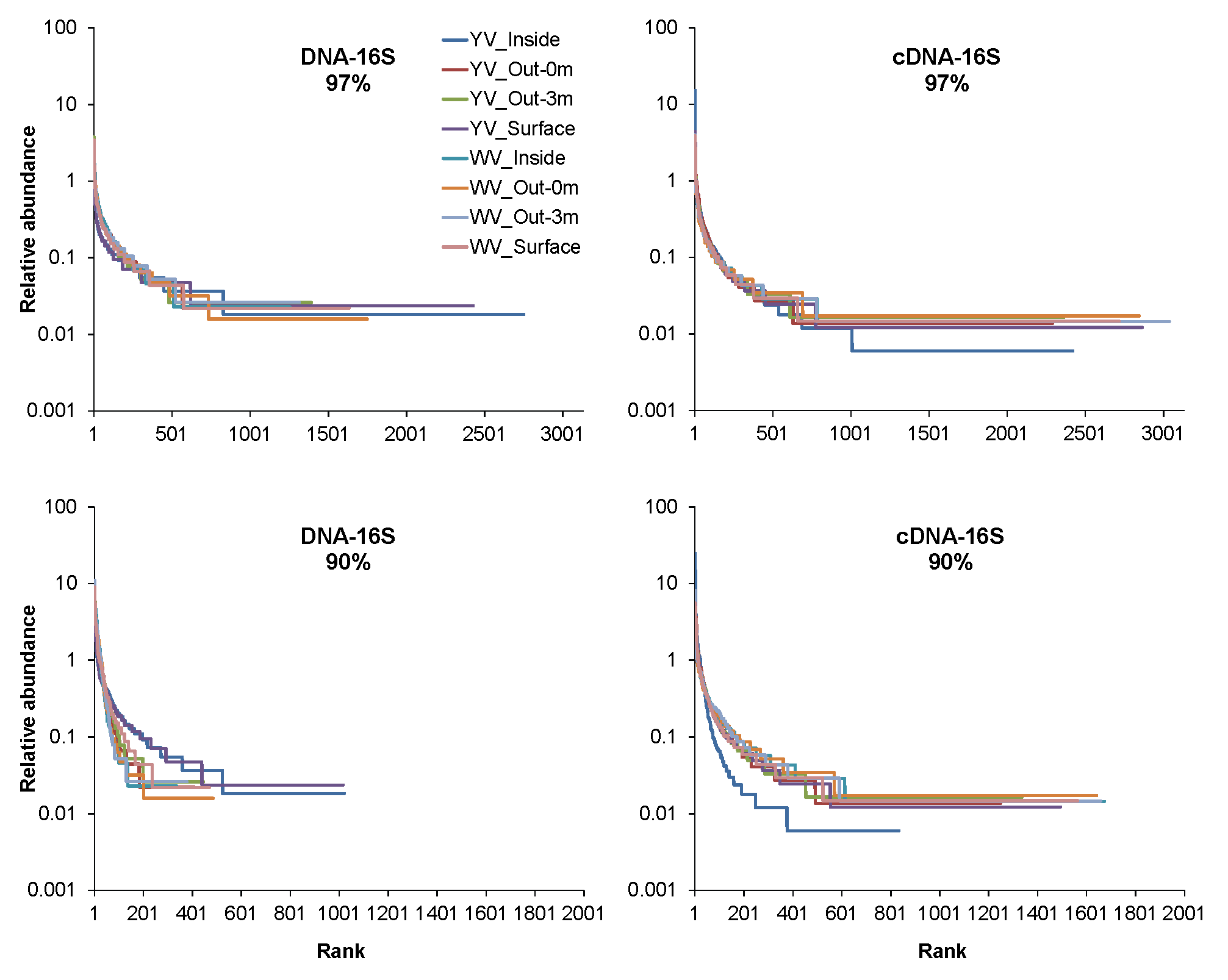

Supplement: Figure S4 — Bacterial rank logarithmic abundance of OTUs at two sequence similarity levels (97% and 90%) in each of DNA- and RNA-based libraries. (TIF) [file pone.0044593.s004.tif]

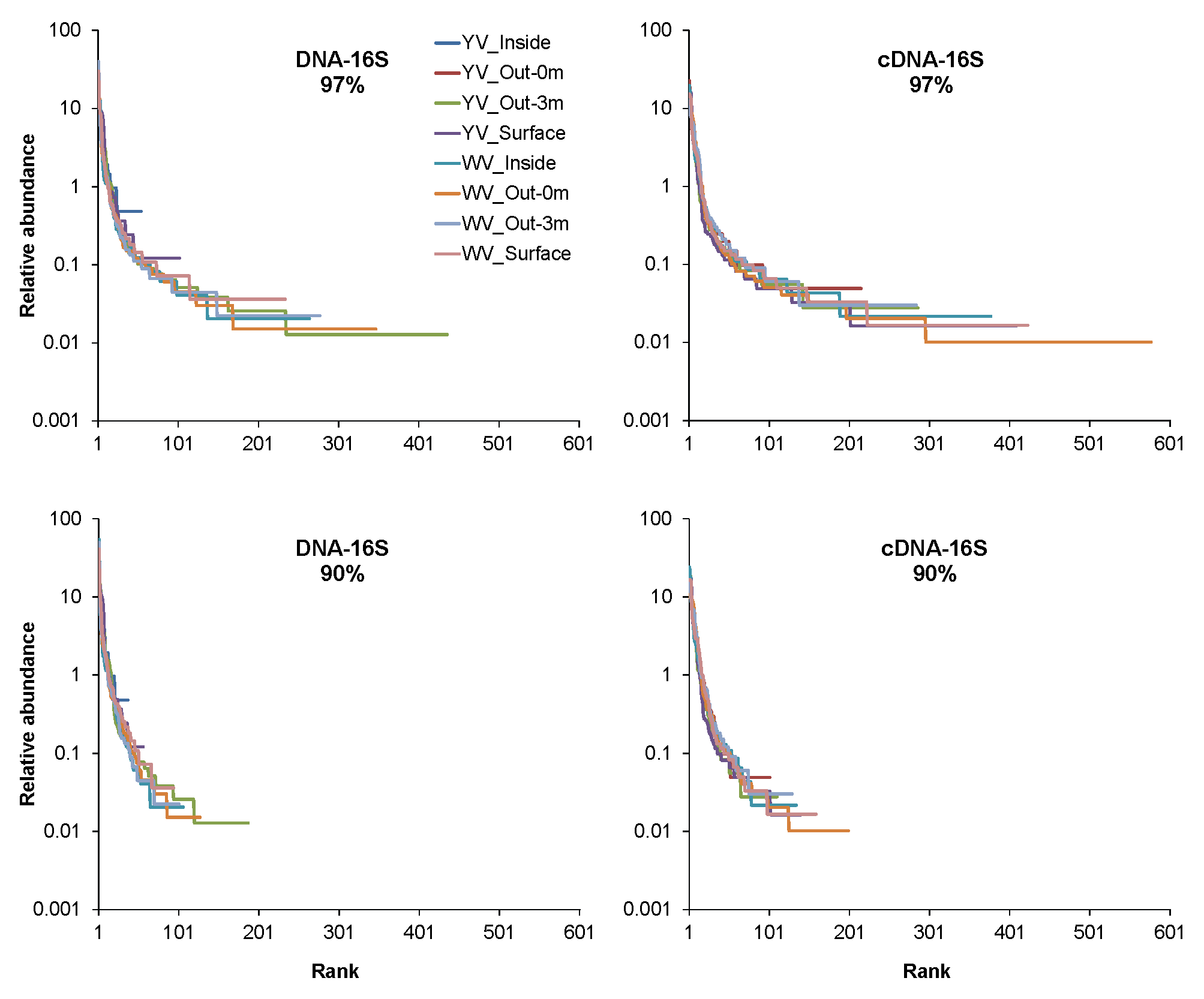

Supplement: Figure S5 — Archaeal rank logarithmic abundance of OTUs at two sequence similarity levels (97% and 90%) in each of DNA- and RNA-based libraries. (TIF) [file pone.0044593.s005.tif]
