# Supplementary material for: Sulfur Metabolizing Microbes Dominate Microbial Communities in Andesite-Hosted Shallow-Sea Hydrothermal Systems
Source: PLoS One. 2012 Sep 7;7(9):e44593. doi: 10.1371/journal.pone.0044593 (PMC3436782; doi:10.1371/journal.pone.0044593)
Supplement: Table S1 — Similarity-based OTUs, species richness and diversity estimates of bacterial communities. (DOC) [file pone.0044593.s006.doc]

**Table S1.** Similarity-based OTUs, species richness and diversity estimates of bacterial communities.

|  | DNA | | | | | | | cDNA | | | | | | |
| --- | --- | --- | --- | --- | --- | --- | --- | --- | --- | --- | --- | --- | --- | --- |
| Sample ID | Trimmed tags | Cutoff | OTUs | Coverage | ACE | Chao1 | Shannon | Trimmed tags | Cutoff | OTUs | Coverage | ACE | Chao1 | Shannon |
| YV_Inside | 5456 | 0.03 | 2,752 | 0.647 | 13,358 | 7,595 | 7.442 | 16731 | 0.03 | 2,421 | 0.915 | 8,716 | 5,518 | 5.751 |
|  |  | 0.05 | 1,880 | 0.798 | 5,544 | 3,892 | 6.801 |  | 0.05 | 1,516 | 0.949 | 5,277 | 3,387 | 5.064 |
|  |  | 0.10 | 1,021 | 0.909 | 2,321 | 1,771 | 5.822 |  | 0.10 | 833 | 0.973 | 2,413 | 1,634 | 3.861 |
| YV_Out-0m | 4508 | 0.03 | 1,444 | 0.805 | 5,324 | 3,290 | 6.471 | 7321 | 0.03 | 2,467 | 0.752 | 16,995 | 8,571 | 6.583 |
|  |  | 0.05 | 868 | 0.894 | 2,705 | 1,758 | 5.651 |  | 0.05 | 2,022 | 0.805 | 11,893 | 6,439 | 6.226 |
|  |  | 0.10 | 407 | 0.950 | 1,342 | 870 | 4.321 |  | 0.10 | 1330 | 0.889 | 4,698 | 3,168 | 5.562 |
| YV_Out-3m | 3837 | 0.03 | 1,392 | 0.762 | 5,918 | 3,680 | 6.434 | 6067 | 0.03 | 2,539 | 0.683 | 19,308 | 9,249 | 6.812 |
|  |  | 0.05 | 899 | 0.861 | 3,333 | 1,919 | 5.637 |  | 0.05 | 2,056 | 0.755 | 13,485 | 6,805 | 6.442 |
|  |  | 0.10 | 446 | 0.935 | 1,624 | 849 | 4.479 |  | 0.10 | 1417 | 0.843 | 6,826 | 4,128 | 5.742 |
| YV_Surface | 4230 | 0.03 | 2,427 | 0.572 | 14,204 | 7,619 | 7.376 | 8182 | 0.03 | 3,070 | 0.719 | 23,062 | 11,372 | 6.837 |
|  |  | 0.05 | 1,783 | 0.719 | 7,561 | 4,328 | 6.818 |  | 0.05 | 2,423 | 0.793 | 14,159 | 7,402 | 6.442 |
|  |  | 0.10 | 1,016 | 0.864 | 3,038 | 2,139 | 5.894 |  | 0.10 | 1590 | 0.874 | 7,020 | 4,237 | 5.668 |
| WV_Inside | 4356 | 0.03 | 1,254 | 0.829 | 4,223 | 2,785 | 6.270 | 6927 | 0.03 | 3,120 | 0.663 | 22,000 | 10,578 | 7.213 |
|  |  | 0.05 | 733 | 0.906 | 2,741 | 1,639 | 5.395 |  | 0.05 | 2,581 | 0.738 | 14,988 | 7,667 | 6.905 |
|  |  | 0.10 | 335 | 0.954 | 1,336 | 853 | 4.026 |  | 0.10 | 1737 | 0.839 | 7,712 | 4,615 | 6.195 |
| WV_Out-0m | 6293 | 0.03 | 1,749 | 0.839 | 5,754 | 3,811 | 6.590 | 5791 | 0.03 | 3,023 | 0.591 | 25,208 | 12,077 | 7.327 |
|  |  | 0.05 | 1,000 | 0.908 | 3,911 | 2,579 | 5.666 |  | 0.05 | 2,507 | 0.685 | 15,850 | 8,149 | 7.014 |
|  |  | 0.10 | 485 | 0.955 | 1,675 | 1,043 | 4.209 |  | 0.10 | 1719 | 0.803 | 8,523 | 4,735 | 6.320 |
| WV_Out-3m | 3812 | 0.03 | 1,314 | 0.792 | 4,481 | 3,049 | 6.495 | 6879 | 0.03 | 3,242 | 0.637 | 26,413 | 12,672 | 7.262 |
|  |  | 0.05 | 743 | 0.890 | 2,613 | 1,710 | 5.584 |  | 0.05 | 2,631 | 0.725 | 15,957 | 8,484 | 6.900 |
|  |  | 0.10 | 378 | 0.935 | 2,634 | 1,003 | 4.150 |  | 0.10 | 1758 | 0.833 | 8,398 | 4,591 | 6.158 |
| WV_Surface | 4567 | 0.03 | 1,634 | 0.768 | 6,744 | 4,190 | 6.585 | 6794 | 0.03 | 2,873 | 0.674 | 25,218 | 11,741 | 6.935 |
|  |  | 0.05 | 1,049 | 0.867 | 3,337 | 2,348 | 5.806 |  | 0.05 | 2,386 | 0.743 | 16,196 | 8,334 | 6.595 |
|  |  | 0.10 | 470 | 0.949 | 1,246 | 854 | 4.587 |  | 0.10 | 1651 | 0.834 | 8,292 | 4,996 | 5.872 |
