# Supplementary material for: Sulfur Metabolizing Microbes Dominate Microbial Communities in Andesite-Hosted Shallow-Sea Hydrothermal Systems
Source: PLoS One. 2012 Sep 7;7(9):e44593. doi: 10.1371/journal.pone.0044593 (PMC3436782; doi:10.1371/journal.pone.0044593)
Supplement: Table S2 — Similarity-based OTUs, species richness and diversity estimates of archaeal communities. (DOC) [file pone.0044593.s007.doc]

**Table S2.** Similarity-based OTUs, species richness and diversity estimates of archaeal communities.

|  | DNA | | | | | | | cDNA | | | | | | |
| --- | --- | --- | --- | --- | --- | --- | --- | --- | --- | --- | --- | --- | --- | --- |
| Sample ID | Trimmed tags | Cutoff | OTUs | Coverage | ACE | Chao1 | Shannon | Trimmed tags | Cutoff | OTUs | Coverage | ACE | Chao1 | Shannon |
| YV_Inside | 208 | 0.03 | 54 | 0.851 | 198 | 106 | 3.220 | - | - | - | - | - | - | - |
|  |  | 0.05 | 50 | 0.880 | 128 | 80 | 3.178 |  | - | - | - | - | - | - |
|  |  | 0.10 | 37 | 0.918 | 80 | 56 | 2.827 |  | - | - | - | - | - | - |
| YV_Out-0m | - | - | - | - | - | - | - | 2028 | 0.03 | 215 | 0.939 | 656 | 422 | 3.343 |
|  |  | - | - | - | - | - | - |  | 0.05 | 185 | 0.951 | 518 | 375 | 3.271 |
|  |  | - | - | - | - | - | - |  | 0.10 | 101 | 0.975 | 344 | 183 | 2.884 |
| YV_Out-3m | 7793 | 0.03 | 436 | 0.974 | 867 | 714 | 3.481 | 3612 | 0.03 | 286 | 0.960 | 690 | 480 | 3.324 |
|  |  | 0.05 | 340 | 0.981 | 682 | 518 | 3.345 |  | 0.05 | 232 | 0.972 | 453 | 347 | 3.244 |
|  |  | 0.10 | 187 | 0.991 | 265 | 271 | 2.859 |  | 0.10 | 110 | 0.987 | 174 | 179 | 2.721 |
| YV_Surface | 825 | 0.03 | 102 | 0.930 | 346 | 252 | 3.207 | 6125 | 0.03 | 409 | 0.966 | 928 | 700 | 3.294 |
|  |  | 0.05 | 92 | 0.939 | 280 | 228 | 3.140 |  | 0.05 | 319 | 0.978 | 486 | 488 | 3.201 |
|  |  | 0.10 | 56 | 0.976 | 79 | 80 | 2.721 |  | 0.10 | 140 | 0.994 | 172 | 166 | 2.721 |
| WV_Inside | 4903 | 0.03 | 264 | 0.974 | 622 | 472 | 2.807 | 4613 | 0.03 | 377 | 0.959 | 834 | 642 | 3.585 |
|  |  | 0.05 | 197 | 0.984 | 363 | 306 | 2.715 |  | 0.05 | 296 | 0.973 | 450 | 430 | 3.478 |
|  |  | 0.10 | 106 | 0.991 | 214 | 172 | 2.044 |  | 0.10 | 134 | 0.988 | 280 | 267 | 2.868 |
| WV_Out-0m | 6621 | 0.03 | 347 | 0.973 | 915 | 686 | 2.925 | 9836 | 0.03 | 577 | 0.971 | 1230 | 973 | 3.713 |
|  |  | 0.05 | 248 | 0.984 | 488 | 444 | 2.809 |  | 0.05 | 448 | 0.981 | 678 | 677 | 3.629 |
|  |  | 0.10 | 127 | 0.994 | 179 | 178 | 2.260 |  | 0.10 | 199 | 0.992 | 364 | 302 | 3.111 |
| WV_Out-3m | 4482 | 0.03 | 277 | 0.971 | 566 | 422 | 2.869 | 3314 | 0.03 | 284 | 0.956 | 745 | 534 | 3.722 |
|  |  | 0.05 | 197 | 0.984 | 274 | 261 | 2.754 |  | 0.05 | 247 | 0.965 | 528 | 411 | 3.654 |
|  |  | 0.10 | 101 | 0.993 | 136 | 124 | 2.167 |  | 0.10 | 129 | 0.983 | 271 | 216 | 3.119 |
| WV_Surface | 2770 | 0.03 | 234 | 0.957 | 601 | 404 | 3.153 | 6034 | 0.03 | 423 | 0.967 | 918 | 687 | 3.617 |
|  |  | 0.05 | 180 | 0.974 | 317 | 249 | 3.039 |  | 0.05 | 334 | 0.978 | 596 | 478 | 3.524 |
|  |  | 0.10 | 94 | 0.990 | 124 | 116 | 2.520 |  | 0.10 | 159 | 0.990 | 289 | 224 | 2.988 |

-, PCR reactions were not successful.
